# Mutation in ESBL Plasmid from *Escherichia coli* O104:H4 Leads Autoagglutination and Enhanced Plasmid Dissemination

**DOI:** 10.3389/fmicb.2018.00130

**Published:** 2018-02-02

**Authors:** Mickaël Poidevin, Mari Sato, Ipek Altinoglu, Manon Delaplace, Chikara Sato, Yoshiharu Yamaichi

**Affiliations:** ^1^Institute for Integrative Biology of the Cell, Université Paris-Saclay, CEA, CNRS, Université Paris-Sud, Gif-sur-Yvette, France; ^2^Biomedical Research Institute, National Institute of Advanced Industrial Science and Technology, Tsukuba, Japan; ^3^Graduate School of Structure and Dynamics of Living Systems, Université Paris-Sud, Orsay, France; ^4^Master of Science and Technology, University Pierre and Marie Curie, Paris, France

**Keywords:** horizontal gene transfer, multidrug resistant, ESBL, conjugation, atmospheric scanning electron microscopy

## Abstract

Conjugative plasmids are one of the main driving force of wide-spreading of multidrug resistance (MDR) bacteria. They are self-transmittable via conjugation as carrying the required set of genes and *cis*-acting DNA locus for direct cell-to-cell transfer. IncI incompatibility plasmids are nowadays often associated with extended-spectrum beta-lactamases producing Enterobacteria in clinic and environment. pESBL-EA11 was isolated from *Escherichia coli* O104:H4 outbreak strain in Germany in 2011. During the previous study identifying transfer genes of pESBL-EA11, it was shown that transposon insertion at certain DNA region of the plasmid, referred to as Hft, resulted in great enhancement of transfer ability. This suggested that genetic modifications can enhance dissemination of MDR plasmids. Such ‘superspreader’ mutations have attracted little attention so far despite their high potential to worsen MDR spreading. Present study aimed to gain our understanding on regulatory elements that involved pESBL transfer. While previous studies of IncI plasmids indicated that immediate downstream gene of Hft, *traA*, is not essential for conjugative transfer, here we showed that overexpression of TraA in host cell elevated transfer rate of pESBL-EA11. Transposon insertion or certain nucleotide substitutions in Hft led strong TraA overexpression which resulted in activation of essential regulator TraB and likely overexpression of conjugative pili. Atmospheric Scanning Electron Microscopy observation suggested that IncI pili are distinct from other types of conjugative pili (such as long filamentous F-type pili) and rather expressed throughout the cell surface. High transfer efficiency in the mutant pESBL-EA11 was involved with hyperpiliation which facilitates cell-to-cell adhesion, including autoagglutination. The capability of plasmids to evolve to highly transmissible mutant is alarming, particularly it might also have adverse effect on host pathogenicity.

## Introduction

Worldwide dissemination of antibiotic resistance (and in many cases, MDR) is one of the most important issues in public health. MDR is often associated with (re-)emerging infectious diseases and epidemics, and in many cases, the determinants for the MDR are encoded on the conjugative plasmid which is capable of its cell-to-cell transmission via conjugation. In the devastated outbreak of enterohemorrhagic/enteroaggregative *Escherichia coli* O104 in Germany (and other European countries) in 2011, the causative strain harbored a plasmid encoding two ESBL genes, pESBL-EA11 (hereafter referred to as pESBL) (Frank et al., 2011; Rasko et al., 2011; Rohde et al., 2011).

pESBL belongs to IncI incompatibility group of plasmid and encodes sufficient set of genes and *cis*-elements for its conjugational transfer, hence it can be transmitted between enterobacteria such as *E. coli* and *Klebsiella pneumoniae* (Yamaichi et al., 2015). Despite the diversity of conjugative plasmids found in natural or clinical environments, fundamental steps of conjugational transfer are conserved among different plasmids. Conjugative (sex) pili exported by MPF systems, also known as T4SS, is required for cell-to-cell contact which eventually fuse membranes or allow DNA transfer through the pili, whereas DNA processing (MOB) systems create a nick at origin of transfer (*oriT*) and subsequently strip a single-stranded DNA for the entire plasmid (Smillie et al., 2010). Essentially, MPF/T4SS and MOB systems can be classified into only a few systems (four and six, respectively), and IncI conjugative plasmids consist MPF_I_ type of MPF/T4SS system (Smillie et al., 2010). In classic prototype of IncI plasmids, R64, transfer genes were identified by brute force approach including knocking out of each gene (Komano et al., 2000 and references therein). By contrast, transfer genes of pESBL have been recently addressed by genome-wide approach, transposon insertion site sequencing (Tnseq) (Yamaichi et al., 2015). Nevertheless, list of transfer genes of these two plasmids are parallel and consists of 4 clusters: *oriT* and *nikAB* genes corresponding to MOB system, *tra/trb* gene cluster for conjugation in general corresponding to MPF/T4SS system, *pil* gene cluster for synthesis of pili, and *traABCD* regulatory gene cluster (of which *traBC* are essential for conjugation, and *traD* is not present in pESBL) (Sampei et al., 2010; Yamaichi et al., 2015). For clarity, genes with same name but in different MPF/T4SS systems do not necessary mean they are homologous (for example, TraA from F plasmid encodes prepropilin and has no similarity to TraA from pESBL).

**FIGURE 1:**
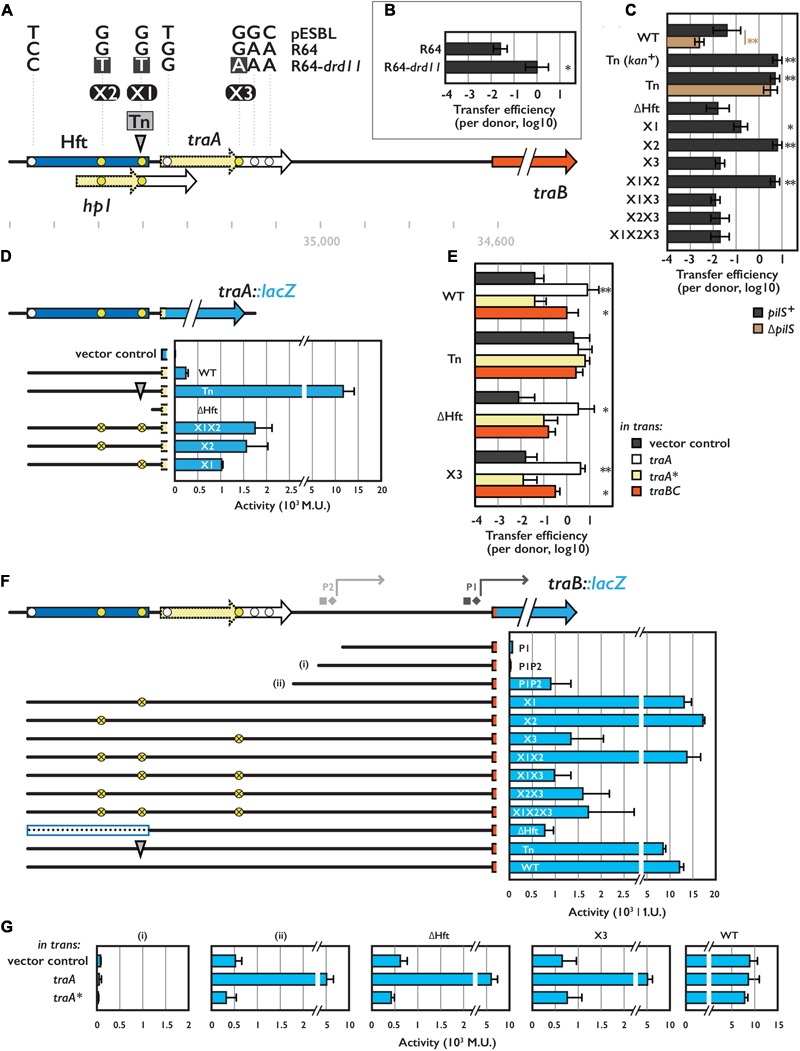
Hft and its flanking region involved in transcriptional regulation for conjugative transfer. **(A)** Schematic representation of the Hft (shown in blue box) and flanking *tra* genes. Arrows with solid and broken line indicate coding sequences and its truncated variants, respectively. Transposon insertion site for the Tn mutant is shown by the gray arrowhead. Single nucleotide polymorphisms among pESBL, R64 and R64-*drd11* are also indicated (see text). Coordinates shown below are obtained from NC_018659.1. **(B)** Transfer efficiencies of R64 and R64-*drd11*. From **(B)** through **(G)**, the mean and standard deviations of at least three independent experiments are shown. **(C,E)** Transfer efficiencies of various pESBL variants. In **(E)**, donor cells harbor an extra plasmid for overexpression of indicated gene. **(D,F,G)** Reporter assay for TraA **(D)** and TraB **(F,G)** with indicated construct. β-galactosidase activities were shown in Miller Unit (M.U.). In **(G)**, cells harbor an extra plasmid for overexpression of indicated gene. In **(F)**, square and diamond indicate predicted –35 and –10 sequence, respectively. ^∗^Indicates *p* < 0.05 and ^∗∗^ indicates *p* < 0.01, respectively.

Remarkably, Tnseq revealed that the short DNA region (dubbed as Hft for high frequency transfer) upstream of *traABC* regulates transfer efficiency of pESBL (**Figure 1A**). Transposon insertion in the region resulted in highly (>10-fold) elevated transfer efficiency (Yamaichi et al., 2015), which is alarming as a simple transposition event can dramatically increase the transmission of already highly transmittable conjugative plasmids. Such ‘superspreader’ mutants could evolve any time in various ways. For instance, widespread plasmid pOXA-48a has a transposon inserted in the *tir* gene, and the disruption of Tir results in elevated transfer efficiency by unknown mechanism (Poirel et al., 2012; Potron et al., 2014). *stbA* mutation in broad-host-range R388 increased transfer efficiency of the plasmid by 50-fold, although exhibiting instability in the host cell in exchange (Guynet et al., 2011). In fact, mutants of resistance plasmids with increased transfer rates have already been described and isolated several decades ago (Meynell and Datta, 1967). As ability of transfer is considered to be repressed by regulatory genes in normal state, these mutants were called ‘derepressed’ and have been widely used in research. *E. coli* fertility factor (F plasmid) which presents high transfer efficiency can be also considered as ‘derepressed’, since it has authentic mutation in *finO* repressor (Frost et al., 1994). As derepressed plasmids exhibit elevated transfer efficiency, they often express more pili (Meynell et al., 1968; Bradley, 1980a), and presumably in a consequence of cell-to-cell adhesion, promote development of biofilm (Ghigo, 2001).

Due to their characteristics, visualization of pili is not easy and has mainly done with Electron Microscopy (EM) using derepressed plasmid but pili were detached from cells followed by enrichment *in vitro*. Antibodies or bacteriophages targets particular conjugative pili were also used for specific labeling. Initial morphological and serological observations by EM in early 80’s classified conjugative pili in 3 classes: thin flexible, thick flexible and rigid (Bradley, 1980a,b, 1983, 1984). Since then, researches have mainly focused on F-type pili (represented by F plasmid) which is flexible and can be extended up to 20 μm (Meynell et al., 1968). IncI plasmids were reported to possess two different kinds of pili: thick and thin ones (Bradley, 1980b, 1983). In R64, *pil* operon, which encodes proteins homologous to type IV pilus, was shown to be involved in liquid but not on solid-surface conjugation (Kim and Komano, 1997; Yoshida et al., 1999). While *pil* genes considered to encode ‘thin’ pilus in R64 (Kim and Komano, 1997; Yoshida et al., 1999), 5 *pil* genes in pESBL are classified as essential for conjugational transfer on solid surface (Yamaichi et al., 2015). Nonetheless, distinguishing the two kinds of pili attached to the host cell has not been successful. P-type conjugative pili (represented by RP4 and R388 plasmids, not confused by P-pili/fimbriae from uropathogenic *E. coli*) were expedited by MPF_T_ type of MPF/T4SS system (Lawley et al., 2003; Smillie et al., 2010). Bradley originally reported them to be shorter and more rigid (Bradley, 1980b), however, controversial morphology was also described from *Agrobacterium tumefaciens* Ti plasmid (Fullner et al., 1996).

Although EM provides sub-nanometer resolution, biological samples require unidealistic pretreatment such as dehydration and under vacuum. Recent innovation of ASEM, however, allows visualization of nanostructures in aqueous solution at atmospheric pressure. In bacteria, extracellular proteins such as pili and flagella, and biofilm matrices have been successfully observed by ASEM using immunogold labeling with specific antibody or rather non-specific labeling with positively and/or negatively charged Nanogold (Sato et al., 2012; Nishiyama et al., 2014; Sugimoto et al., 2016).

## Materials and Methods

### Plasmids and Strains

Plasmids and strains used in this study were listed in Supplementary Tables S1, S2, respectively.

*kan* gene cassette from pESBL::Tn1 transposon insertion mutant (Yamaichi et al., 2015) was removed by *in vivo* expression of FRT recombinase using pCP20 (Datsenko and Wanner, 2000; Chiang and Rubin, 2002).

*ΔfliA* mutation was introduced by P1 transduction from JW1907 (KEIO collection, Baba et al., 2006), and the mutation was confirmed by antibiotic resistance and motility phenotype.

Gene deletions (*ΔoriT* and *ΔpilS*) and introduction of point mutation(s) in pESBL were carried out by conventional double-crossovers with pDM4-based plasmids as previously described (Milton et al., 1996; Yamaichi et al., 2015). To introduce point mutation(s) in the Hft region, Hft and its flanking regions were amplified and cloned into pDM4 vector, resulting in pEYY39. Nucleotide substitutions were subsequently introduced by QuickChange II XL site-directed mutagenesis kit (Agilent Tech, Santa Clara, CA, United States). In some cases, two allelic exchanges were performed to facilitate introducing and following validation of point mutation(s). Essentially, the Hft region was first replaced by a gene cassette containing *kan* and *rpsL^+^* which screened by resistance to Kanamycin, then second allelic exchange to introduce point mutation(s) were selected by resistance to Streptomycin. *kan*-*rpsL^+^* gene cassette, which was amplified with primers oYo417 and oYo421, was inserted in pEYY39 *in vivo* by λ Red recombination technique (Datsenko and Wanner, 2000). Gene replacements in pESBL were carried out by conventional double-crossovers as previously described (Yamaichi et al., 2015). For the second step of the two steps replacement strategy, β2163 (Demarre et al., 2005) was used for the donor instead of conventional SM10 λ *pir* so that exconjugants can be selected by growth on LB plate without diammonium phosphate. Inherited ability of conjugative transfer was used to move pESBL and its derivatives between different *E. coli* strains.

For the cloning of plasmids for β-galactosidase assay, DNA fragments were amplified from corresponding pESBL mutant and cloned into pCB192-YY (Yamaichi et al., 2011) linearized by *Eco*RI-*Hin*dIII, either by conventional restriction cloning or Gibson Assembly (Gibson et al., 2009).

Unless specified, clonings were done by Gibson Assembly. When necessary, nucleotide sequence was verified by Sanger sequencing (GATC biotech, Germany). oYo170 and oYo171 were used for sequencing of Hft and its flanking region of R64. Oligo nucleotides used in this study were listed in Supplementary Table S3.

### Cell Growth

Otherwise specified, cells were grown in LB in liquid or on agar (1.5%) plate at 37°C. Supplements were used in following concentrations when appropriate; Ampicillin 100 μg/mL, Chloramphenicol 25 μg/mL, Kanamycin 25 μg/mL, Streptomycin 100 μg/mL, Tetracycline 10 μg/mL, Arabinose 0.1%, diammonium phosphate 300 μM.

To measure growth rate, overnight culture of cells were backdiluted in 50 mL of LB in 250-mL Erlenmeyer and grown at 37°C with orbital shaking at 180 rpm. OD 600 nm and colony forming units were measured every 30–60 min of incubation. Alternatively, a microplate reader (Tecan Infinite M200 Pro, Tecan Group, Switzerland) was used. In a conventional flat-bottom, 96-well cell culture plate, 150 μL of LB medium was aliquoted then the overnight culture was inoculated at 1:500 dilution. OD 600 nm was measured every 15 min during the 12-h growth at 37°C with orbital shaking (4 mm pitch). For each experiment, samples were prepared in triplicate.

To measure sedimentation, 25-mL overnight culture in a 100-mL Erlenmeyer was settled at room temperature and 100∼1000 μL of supernatant was carefully taken for the measurement of OD 600 nm every 1 h.

To measure mobility/chemotaxis, overnight culture was diluted 10 times then 1 μL was inoculated in LB motility plate containing 0.3% agar. After incubating at room temperature for overnight, X and Y diameters of the zone cells expanded were measured. For the reference, each test plate included parental *E. coli* strain without plasmid.

Susceptibility to β-lactam antibiotics were tested by ETEST (bioMérieux) with supplier’s instruction.

### Bacterial Conjugation and Transfer Efficiency

For the conventional surface mating, 100 and 10 μL of overnight cultures of recipient and donor cells, respectively, were washed once with LB to remove antibiotics then mixed and spun down in Eppendorf tube. Cells were then resuspended in 50 μL of LB and placed on a 0.45-μm HAWP filter (EMD Milipore, Billerica, MA, United States) on LB agar plate. After incubation at 37°C for 2 h, cells were recovered in 1 mL of LB in a 50-mL centrifuge tube then plated on LB agar plates containing relevant antibiotics with appropriate dilutions.

For ‘snap conjugation’ assay, overnight cultures of recipient and donor cells were backdiluted and grown for 2.5 h without antibiotic selection. No loss of pESBL or F plasmid was detected after the 2.5 h of growth. In an Eppendorf tube, 890 μL of LB, 100 μL of recipient cells and 10 μL of donor cells were added sequentially. The tube was immediately vortexed for 3 s followed by plating the cells on LB agar containing appropriate antibiotics.

Transfer efficiencies were log_10_ transformed then the average and standard deviations were calculated. Student’s *t*-test (heteroscedastic test with two-tailed distribution) was performed in Microsoft Excel for significance analysis.

### β-galactosidase Assay

Assays were performed as previously described (Miller, 1992). A microplate reader (Tecan) was used to measure OD 420, 550, and 600 nm.

### Biofilm Assay

Cells were allowed to form biofilm in LB or M9 medium supplemented with glucose (0.2%) and casamino acid (0.1%), in a flat-bottom 96-well cell culture plate. After 24 h of incubation at 37°C without agitation, biofilms were stained by crystal violet (Sigma–Aldrich, St. Louis, MO, United States) followed by optical quantification (Tecan) as previously described (O’Toole, 2011). *Pseudomonas aeruginosa* strain (PA14) was used for the positive control and the standard. Each sample used 3–4 replicate wells and was examined by at least 3 independent experiments.

### ASEM

*ΔfliA* mutants harboring WT or mutant pESBL were grown in LB medium without agitation then gently placed on poly-L-lysine (Sigma–Aldrich) coated ASEM dish with 8 SiN film windows (Memtily et al., 2015). After 10 min of incubation, cells were fixed by fixing solution (2.5% glutaraldehyde and 1% paraformaldehyde in 0.1 M phosphate buffer pH 7.4) for 15 min. Unattached cells were flushed by miliQ water then the dish was filled by phosphate-buffered saline for transport and storage. The bacterial cells were labeled and observed as described (Nishiyama et al., 2014; Sugimoto et al., 2016). In brief, for the charged Nanogold-labeling, bacteria on the ASEM dish were incubated with 6 μM positively charged 1.4 nm Nanogold solution (Nanoprobes, Yaphank, NY, United States) for 20 min at room temperature. After washing with double-distilled water, the size of the gold particles was increased by gold enhancement using GoldEnhance-EM (Nanoprobes) for 10 min at room temperature, followed by washing with double-distilled water. Bacterial cells immersed in 10 mg/mL ascorbic acid were imaged by ASEM at an acceleration voltage of 20 kV with × 20,000 magnification using backscattered electrons. The electron dose was 6 e^-^/Å^2^, which is 13% of the dose permitted in low-dose cryo-EM aiming at atomic-resolution single-particle reconstructions.

Intensity profile was measured with ‘Plot Profile’ function in ImageJ version 1.51k (Schneider et al., 2012). Fixed length (200 pixels) perpendicular to the long axis was taken approximately one quarter of the cell length position and each profile was recorded by ‘ROI Manager’ in ImageJ. Analysis of data including subtraction of the background intensity and graph drawing was done with MATLAB version 2013a with Statistics and Machine Learning Toolbox (MathWorks, Natick, MA, United States).

### Field Emission Scanning EM (FE-SEM)

Traditional Pt-coated FE-SEM was performed by modification of the method described in Nishiyama et al. (2014). Briefly, *E. coli* cells were cultured on SiN layer on Si chip, fixed by the fixing solution at room temperature for 15 min, washed with phosphate buffer, dehydrated with alcohol gradient series, and dried using the critical point drying technique (Leica CPD300, Leica Camera, Germany). The cells were sputter coated with platinum (∼3 nm thickness) (Quick cool coater SC701MC, Sanyu Co., Ltd., Japan), and observed with a FE-SEM, JSM 7400F (JEOL Ltd., Japan) using secondary electrons. The acceleration voltage of the FE-SEM was 1.5 kV and the working distance was 8 mm.

## Results

### Comparison of the Hft Region in Different Plasmids and Their Superspreader Derivatives

To address the regulation of conjugational transfer by the Hft region in pESBL, we first engineered a superspreader mutant pESBL::Tn1 isolated in the previous work (Yamaichi et al., 2015). Using Flp-*FRT* site-specific recombination, kanamycin resistant cassette was removed which resulted in 199 bp scar inserted in the Hft region flanked with duplication of TA dinucleotide. While the resulting plasmid (hereafter referred to as Tn mutant) is now sensitive to kanamycin and eliminated potential polar effect, it retained all the phenotypes including elevated transfer efficiency from parental pESBL::Tn1 (**Figures 1A,C** and Supplementary Figures S1A,C).

Next, we tried to determine nucleotide substitution(s) that can elevate transfer efficiency. To do so, we compared the nucleotide sequence of the Hft region to the similar IncI conjugative plasmid, R64. In fact, the R64 sequence registered in the Genbank (AP005147.1, Sampei et al., 2010) is not *bona fide* R64, but a derivative *drd11* which presents derepressed/superspreader phenotype. Enhancement of transfer efficiencies from pESBL WT to Tn mutant and from R64 to R64 *drd11* were somewhat similar (**Figures 1B,C**). Therefore, we sequenced the ∼600 bp region encompassing Hft and its downstream *traA* gene of the WT and *drd11* of R64. To this end, we identified seven mismatched bases among three plasmids of which three were located in the Hft region. Particular interest was gathered in three of the seven mismatches that are specific to R64-*drd11*, while the other four appeared to be divergence between pESBL and R64 (**Figure 1A**, yellow and white circles, respectively). To elucidate the impact of the three mutations highlighted in R64-*drd11*, nucleotide substitutions (dubbed X1, X2, and X3, see **Figure 1A**) were introduced in pESBL and resulting mutant plasmids were subjected to conjugational transfer experiments. As shown in **Figure 1C**, X2 showed increased transfer efficiency that was comparable to the Tn mutant. In contrast, X3 exhibited rather decreased transfer efficiency. Furthermore, X3 mutation was epistatic to X2: neither X2X3 double nor X1X2X3 triple mutant showed increased transfer efficiency (**Figure 1C**). These results suggest that not only transposition but also nucleotide substitution in the Hft region can result in enhanced conjugational transfer. Even though they are very similar, pESBL X1X2X3 triple mutant did not show elevated transfer efficiency as seen in R64-*drd11*. Thus it is possible that pESBL and R64 may not possess exact regulatory mechanism, or R64-*drd11* includes further mutations than the three nucleotide substitutions to manifest its higher transfer ability. This suggests that there could be multiple ways for plasmid to evolve to superspreader.

### Regulators of Conjugational Transfer of pESBL

In the Hft region, there is an open reading frame encoding hypothetical protein Hp1, and X1 and Tn harbor mutation in its coding region (**Figure 1A**). Lines of evidence suggested that Hp1 protein does not involve in conjugational transfer. Notably, overexpression of Hp1 *in trans* in the donor cell did not affect transfer efficiency of pESBL (data not shown).

Besides X1/Tn disrupting Hp1, nucleotide substitution at X3 position results in truncation of TraA protein (hereafter truncated TraA will be denoted as TraA^∗^). Previous results showed that neither *traA* of pESBL nor *traA*^∗^ of R64-*drd11* is essential for conjugation (Kim et al., 1993; Yamaichi et al., 2015). However, we found that overexpression of TraA in the donor cell increased the transfer efficiency of WT pESBL comparable to the Tn mutant (**Figure 1E**). Consistent to the transfer efficiency assay, transposon insertion at Hft resulted in huge (∼50-fold) increase of *traA::lacZ* expression level compared to the WT context. On the other hand, deletion of the entire Hft region abolished the expression of *traA::lacZ* (**Figure 1D**). Providing intact TraA *in trans* in ΔHft or X3 pESBL rescued the transfer efficiency that is comparable to the WT pESBL with TraA overexpression (**Figure 1E**). These results indicated that TraA is directly or indirectly involved in pESBL transfer.

In contrast to *traA* and *traA^∗^*, downstream *traBC* genes were previously classified as essential for transfer in both pESBL and R64-*drd11* (Kim et al., 1993; Yamaichi et al., 2015). When TraBC was overexpressed *in trans* in the donor cell, enhanced transfer efficiency of pESBL was also observed (**Figure 1E**). It is noteworthy that extent of the enhancement of transfer efficiency by TraBC overexpression was somewhat lower than by overexpression of TraA. Particularly in case of Tn and ΔHft, there were no significant increase compared to the vector control (*p*-value 0.71 and 0.07, respectively) (**Figure 1E**). Next, we investigated *traBC* expression level. Even though two potential promoters (P1 and P2 in **Figure 1F**) for *traB* were predicted by BPROM^1^, these two sites were not sufficient for *traB::lacZ* expression (**Figure 1F**). Considerable expression of TraB::LacZ was observed when additional 54 bp upstream to P2 was included, but it was significantly increased when the reporter plasmid contained the Hft region (**Figure 1F**). Deletion of Hft or introduction of X3 nucleotide substitution in the reporter plasmid alleviated the enhancement, suggesting that TraA could activate TraB expression. Consistent to the idea, expression of *traA in trans* boosted the TraB::LacZ expression when the reporter plasmid contains additional 54 bp upstream to the P2 (**Figure 1G**, ii). Since TraA did not affect the transcription level of P1P2 context (**Figure 1G**, i), this 54 bp region would contain essential regulatory elements of *traBC* expression. Unlike TraA::LacZ expression, significant increment of TraB::LacZ expression level was not observed in many of Tn, and X1 and/or X2 constructs, even though they remained to show very high (**Figure 1F**). Because these assays were based on high-copy-number plasmid, it is possible that TraA expression was saturated. We also tested whether truncated TraA^∗^ retains functionality or not. TraA^∗^ overexpression appeared to have no effect on both transfer efficiency of WT pESBL and TraB::LacZ expression (**Figures 1E,G**). Therefore, TraA^∗^ is not likely functional at least for the TraB activation.

### Other Phenotypes of the Tn Mutant

**FIGURE 2:**
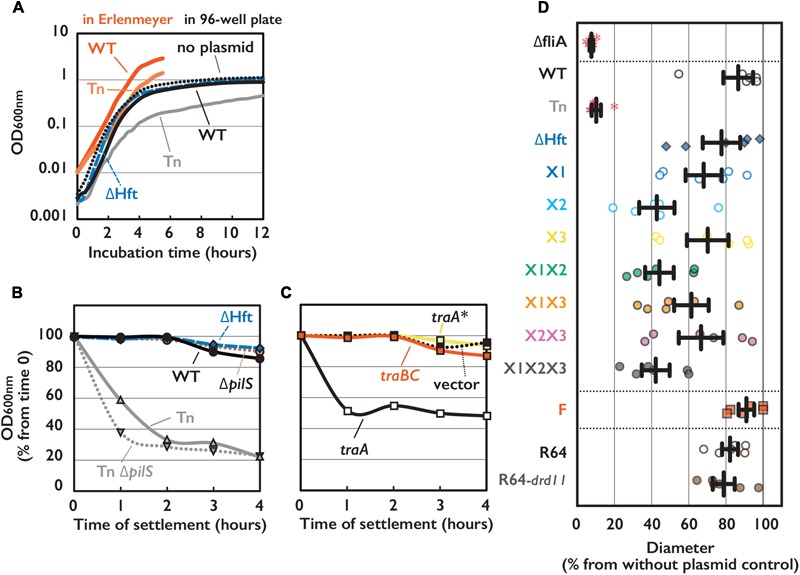
Phenotypes of *E. coli* cells harboring conjugative plasmid. **(A)** Cell growth of *E. coli* strain harboring indicated plasmid measured by OD 600 nm with either conventional spectrometer or 96-well plate reader. **(B,C)** Sedimentation phenotype measured by OD 600 nm of culture supernatant settled on bench for indicated time. *E. coli* cells harboring WT or mutant of pESBL as indicated **(B)** or WT pESBL and an extra plasmid indicated for overexpression **(C)**. Average of at least 3 independent experiments were shown. **(D)** Motility phenotype measured by diameter of expansion on soft agar plate. Results of 6 independent experiments along with the mean and standard deviations were shown. *ΔfliA* mutant without plasmid was used for the negative control. Red asterisks indicate no motility.

*Escherichia coli* harboring the Tn mutant showed other phenotypes than elevated transfer efficiency including growth defect and sedimentation. While harboring WT pESBL did not cause significant difference in growth rate, Tn mutant showed moderate growth defect (**Figure 2A**). It is noteworthy that this growth defect can be overrated when growth rate was measured by OD in an automated plate reader (**Figure 2A**), presumably because of sedimentation phenotype which will be discussed below. When the overnight cultures of *E. coli* harboring different pESBL was settled on the bench, sedimentation of cells were apparent in the Tn mutant, while harboring WT or ΔHft pESBL did not cause host *E. coli* to sediment over the 4 h of time-course experiments (**Figure 2B**). Furthermore, *E. coli* cells harboring Tn mutant was found to be particularly non-motile in motility plate assay (**Figure 2D**) as well as under microscope (data not shown).

It is known that *Vibrio cholerae* culture autoaggultinates when the expression of toxin-coregulated pili was induced (Taylor et al., 1987), and many non-motile *V. cholerae* mutants exhibited the toxin-coregulated pili overexpressed (Gardel and Mekalanos, 1996). Taking them into account, we hypothesized that this sedimentation/autoagglutination phenotype is reflecting the increased cell-to-cell contact which is caused by overexpression of (conjugative) pili encoded on pESBL.

### Altered Cell Surfaces by pESBL

**FIGURE 3:**
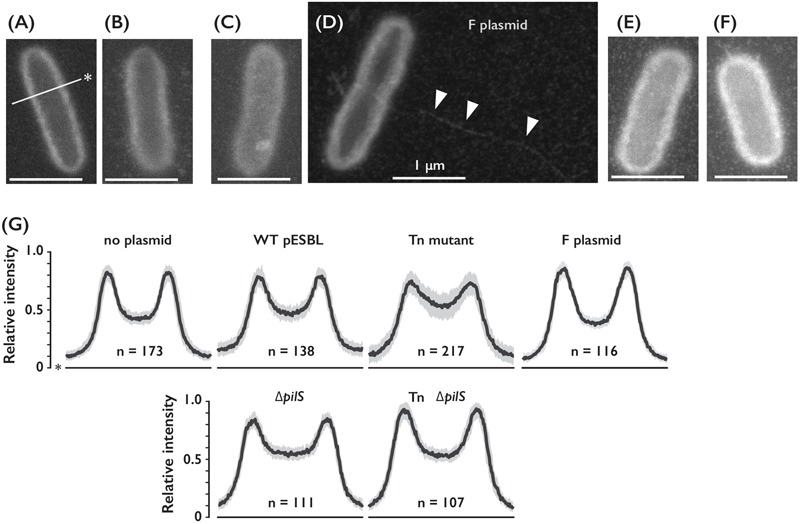
Cell surfaces of *E. coli* cells harboring different conjugative plasmids. **(A–D)** Representative ASEM images of *E. coli* cells without plasmid **(A)**, or harboring either WT **(B)**, Tn **(C)** *ΔpilS* **(E)** or Tn *ΔpilS* mutant **(F)** of pESBL, and F plasmid **(D)**. Arrowheads indicate presumable F pilus. Line with an asterisk indicates perpendicular axis for the intensity profile analysis. Bar = 1 μm. **(G)** Intensity profile of *E. coli* cells with or without plasmids as indicated. Among number of cells analyzed shown as n, median was drawn by the solid line and the gray area represents between the first and third quartiles.

Enhanced cell-to-cell attachment in the Tn mutant was also evident under microscopes as clumps, however, visualization of conjugative pili of pESBL that are associated with *E. coli* cells has been hardly successful. First we transferred test plasmids into *ΔfliA* mutant of *E. coli* (Macnab, 1996) to avoid contamination of flagella. The Tn mutant in *ΔfliA* host showed comparable phenotypes as in the flagellated host, including elevated transfer efficiency and sedimentation (Supplementary Figures S1A,E). Then we carried out experiments to observe pilliation by different plasmids. Our microscopic investigations included traditional FE-SEM with Pt-coated samples. Any particular structures associated to the WT or Tn mutant pESBL samples were not observed in multiple examinations (Supplementary Figure S1F). Therefore, we concluded that conjugative pili would not have been preserved during the sample preparation including dehydrating and vacuum procedures. To overcome potential sample preparation issues, we sought ASEM. In ASEM, aldehyde-fixed cells were treated with positively charged Nanogold particles, which means that negatively charged extracellular structures will be visualized. Somewhat similar to the observation of bacterial cells under fluorescent microscope with membrane dye such as FM4-64, the perimeter of the cells showed highest signal intensities and lesser signals were observed inside the outline, or cell surfaces (**Figure 3**). While we did not see any extracellular structures from negative control cells without plasmid, thin and long filamentous structure was readily detected from cells harboring the F plasmid (**Figures 3A,D**). As this resembled well-known F pili (Brinton et al., 1964; Clarke et al., 2008), we believe that sample preparation for ASEM was capable to maintain conjugative pili attached to the cell. Yet, we did not see any appendages from cells harboring either WT or Tn mutant of pESBL. However, we noticed that they showed different intensities at the cell surfaces (**Figure 3**). To clarify the difference, perpendicular intensity profiles were obtained from more than 100 cells from each sample. As shown in **Figure 3G**, *E. coli* cells harboring WT pESBL showed increased intensity in the cell body, and this was more profound in case of cells possessing the Tn mutant. In contrast, harboring F plasmid did not appear to alter the intensity profile from without plasmid control. These results suggest that pESBL, and possibly other IncI plasmids, exhibit their conjugative pili in a distinct manner to previously described F- and P-types of pili (Christie, 2014). Early EM works by Bradley illustrated pili from IncI plasmids as thick rigid and thin pili (Bradley, 1980b, 1983).

To distinguish plausible thick and thin pili, we deleted *pilS* gene which encodes major subunit of Type IV pili (Kim and Komano, 1997; Horiuchi and Komano, 1998) in WT and Tn pESBL. Increase of intensities in the cell body of *E. coli* cells harboring Tn pESBL was alleviated by introduction of *ΔpilS* (**Figures 3E–G**). Yet we are unsuccessful to gain evidence that two different kinds of pili are appended from the cell, it is suggested that Type IV pili contributed major part of extracellular structures.

### Autoagglutination Phenotype May Facilitate Conjugation in Liquid

**FIGURE 4:**
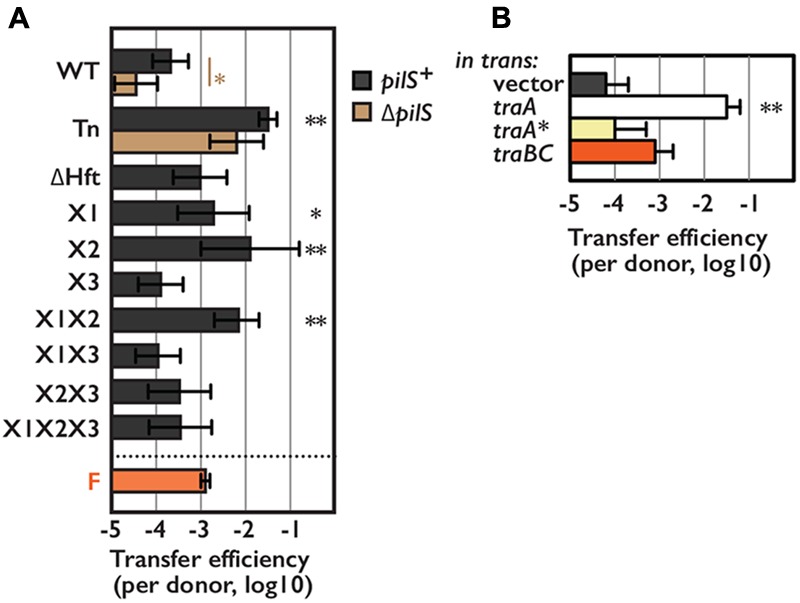
Transfer efficiency of pESBL in snap conjugation. Transfer efficiencies of various pESBL variants **(A)** or WT pESBL harboring an extra plasmid for overexpression of indicated gene **(B)**. F plasmid was used for comparison. Average and standard deviation of at least 3 independent experiments were shown. ^∗^Indicates *p* < 0.05 and ^∗∗^ indicates *p* < 0.01, respectively.

Although Tn mutants of pESBL and F plasmid led marked difference in host *E. coli* cells, both showed very high transfer ability. ASEM results suggested that pESBL does not likely produce long pili that believed to facilitate cell-to-cell attachment. Furthermore, in our transfer efficiency assay conjugation events were occurred on solid surfaces in which cell-to-cell contacts were rather enforced. In some IncI plasmids, Type IV pili were shown to contribute conjugative transfer in liquid condition (Kim and Komano, 1997; Yoshida et al., 1999; Dudley et al., 2006). These made us wondered to test transfer efficiency of pESBL mutants in liquid condition. **Figure 4** shows transfer efficiency in liquid, and in particular, donor and recipient cells were mixed only a few seconds before plating to isolate exconjugants. Tn mutant of pESBL remained to show extreme efficiency for conjugational transfer in this ‘snap conjugation’ condition, and notably it was about 10 times better than the transfer efficiency of the F plasmid. Deletion of *pilS* weaken the transfer efficiency of WT pESBL significantly but the effect was modest and not significant in Tn background (**Figure 4A**). Furthermore, *E. coli* cells harboring *Δ*Tn *pilS* pESBL remained to show sedimentation phenotype (**Figure 2B**). Very efficient cell-to-cell attachment is likely one of the biggest factor to endorse high transfer efficiency of the Tn mutant of pESBL. Interestingly, while the Tn mutant induced cells to autoaggregate and sediment, it does not well promote cells to develop biofilm. In our biofilm assay, only moderate and inconstant biofilm formation was observed with Tn mutant grown in M9 media, and biofilm was barely detected with WT pESBL or when cells were grown in LB (Supplementary Figure S1G). On the other hand, F plasmid does not induce cells to sediment (Supplementary Figure S1D) but was shown to induce formation of thick biofilm (Ghigo, 2001).

## Discussion

Here we showed that TraA as a new positive regulator for conjugational transfer of pESBL. TraA exceeded TraBC in enhancement of transfer efficiency when overexpressed (**Figures 1E, 4B**). Furthermore, TraA, but not TraBC overexpression induced WT pESBL harboring cells to sediment (**Figure 2C**). These lines of evidence suggested that some transfer genes are under control of TraBC, and TraA could regulate not only *traBC* but also its own downstream genes. *traA* was not identified as essential gene for conjugation in previous Tnseq analysis (Yamaichi et al., 2015), probably because expression of essential transfer factor TraB is not fully dependent on TraA. Alternatively, it is possible that *traB* expression level could be retained by polar effect of transposon insertion at *traA*. On the other hand, R64-*drd11* possesses silent mutation in *traA* thus expresses TraA^∗^ rather than intact TraA. Supplying TraA^∗^ did not show significant effect neither on transfer efficiency nor *traB* expression, it is reasonable that inactivation of *traA*^∗^ did not have effect on transfer efficiency in previous study on R64 (Kim et al., 1993).

It is still elusive for full understanding of the regulation mechanism for conjugational transfer. Most puzzling thing is that X1X2 mutations led TraA to be overexpressed, but X3 mutation resulted in expression of truncated TraA^∗^. Yet, R64-*drd11* showed superspreader phenotype in contrast to corresponding pESBL X1X2X3 which presented even little lower transfer efficiency than the WT. Furthermore, sedimentation phenotypes in different point mutants of pESBL as well as R64 variants were not fully explained by TraA and TraBC regulon (Supplementary Figures S1B,D). Protein folding and topology predictions (Käll et al., 2004; Kelley et al., 2015) suggested that TraA has structural similarity to transcriptional regulators. It is also predicted that the C-terminal of the TraA possibly include transmembrane domain, thus TraA and TraA^∗^ might show different protein localization in the cell. So far we do not have direct evidence that TraA regulates transcription of *traBC* for example binding to the upstream 54 bp region. Besides, our preliminary results with *Δhfq E. coli* host suggested involvement of small RNAs in multiple steps including expression of *traA* as well as *traBC* (data not shown). Biochemistry and cell biology of TraA variants are awaiting.

In addition to the Hft∼*traABC* regulation, there could be other layer(s) of regulatory circuit to control transfer efficiency of pESBL. One of such mechanism would be DNA processing that is also an important step for successful conjugational transfer. For instance *ΔoriT* totally abolished the transfer efficiency of pESBL (data not shown), yet Tn *ΔoriT* double mutant showed sedimentation phenotype (Supplementary Figure S1A). Another possible mechanism includes small RNA, and they are scope of future investigations.

In combination with positively charged Nanogold-labeling, ASEM was able to visualize delicate hydrophilic nano-structures on *E. coli* cells (**Figure 3**), and the required pretreatment is just aldehyde fixation and staining. The pili-like structure which was labeled on a small population of *E. coli* cells using positively charged Nanogold (Nishiyama et al., 2014) is similar to the protrusions on cells with F plasmid (**Figure 3D**), and it could be F-type Pili. These results suggest the applicability of this cutting-edge technique to diverse bacterial filaments, as shown for flagella (Nishiyama et al., 2014; Sugimoto et al., 2016). As ASEM also allowed visualization of various eukaryotic cells and host-microbe interaction of mouse stomach (Nishiyama et al., 2010, 2014; Maruyama et al., 2012; Memtily et al., 2015), it could be useful to study the roles and mechanisms for microbial appendages such as pili and flagella interaction with both prokaryotic and eukaryotic cells.

Studies of *pil* operon in R64 suggested that it encodes thin pili that is predicted to belong to the type IVB family (Kim and Komano, 1997). *pilS* deletion in WT pESBL resulted in significant reduction of transfer efficiency in both liquid and solid conjugation. However, in superspreader pESBL Tn background the effect was modest (**Figures 1C, 4A**). It is possible that thin pili is also required for solid conjugation in the WT and not derepressed mutant of R64. Nevertheless, our data suggest that pESBL, and potentially other IncI conjugative plasmids, do not exhibit long filamentous structure but rather possess distinct class of pili that resemble to ‘Velcro.’ Since Δ*pilS* also did not affect sedimentation phenotype of pESBL Tn, ‘thick’ conjugative pili could be sufficient for cell-to-cell adhesion and collision of cells seems enough to establish MPF and following conjugational transfer of the DNA. This characteristic could account for superspreader pESBL mutant’s higher transfer efficiency than F plasmid in snap conjugation. It could also allow mutant pESBL to have fitness advantage in certain niches for the expansion of the plasmid, although transforming cells non-motile and reducing the growth rate. Furthermore, some enteroaggregative *E. coli* strains are shown not to possess canonical genes for its characteristic aggregative adherence. Instead, an IncI plasmid harbored in a such atypical enteroaggregative *E. coli* strain was shown to contribute cell adhesion (Dudley et al., 2006). Thus, it is possible that IncI plasmids not only provide MDR but also enhance host cell ability to adhere which in turn associate to the pathogenicity of the host cell.

Considering the less potency to induce biofilm, it is possible that pESBL pili are more specialized to bacteria-to-bacteria interaction. R64, pESBL, and other IncI plasmids possess shufflon, site-specific recombination mechanism supposed to create variability on the tip of pili (Komano, 1999). Shufflon has been proposed to alter the specificity of recipient in liquid conjugation (Komano et al., 1994), and in R64 and another IncI plasmid ColIb-P9, certain version of PilV shown to be involved in aggregation phenotype (Yoshida et al., 1998). Our preliminary results suggested that *rci* which encodes shufflon-specific DNA recombinase was much higher expressed in Tn and X1X2 mutants than in WT pESBL (data not shown). It is intriguing to see if each of the cells harboring superspreader mutant of pESBL exhibit wider variety of ‘crochet’ to achieve cell-to-cell attachment. Closely related pESBL-305 isolated from chicken cecum has transposon inserted in the shufflon region (Brouwer et al., 2014), although biological consequences are not understood thus far.

Lastly, resistance to antimicrobial reagents is arguably the most important phenotype pESBL provides to the host cell. Even though Tn and ΔHft mutation resulted in increased and decreased transcripts of the two β-lactamase genes, respectively (data not shown), these mutations did not alter minimum inhibitory concentrations of β-lactams we tested; all WT, Tn and ΔHft pESBL led cells resistant against > 256 μg/mL of Amoxicillin and >32 μg/mL of Cefotaxime. It is still possible that these mutations could modify susceptibility to other β-lactam antibiotics.

Altogether, here we showed that mutations in Hft can lead strong TraA overexpression which resulted in overexpression of conjugative pili. Subsequently, hyperpiliation facilitates cell-to-cell adhesion thus promote plasmid transfer efficiency. Potential emergence of superspreader mutant plasmids is threatening, particularly it could make adverse consequence in terms of epidemic of host pathogen. Further investigations could shed light on curbing devastating dissemination of MDR.

## Author Contributions

YY conceived and designed the overall research project. Molecular and genetic experiments were performed by MP, MD, and YY. Biochemical experiments were performed by MP. Microscopy experiments were carried out by MS, CS, and YY, and following image analysis was done by IA. All authors contributed to analyzing data and editing the manuscript. The paper was written by CS and YY.

## Conflict of Interest Statement

The authors declare that the research was conducted in the absence of any commercial or financial relationships that could be construed as a potential conflict of interest.

## Footnotes

1 http://www.softberry.com

## Abbreviations

ASEM: atmospheric scanning electron microscopy
ESBL: extended-spectrum β-lactamase
FE-SEM: field emission scanning electron microscopy
Hft: high frequency transfer
LB: lysogeny broth
M.U.: Miller unit
MDR: multidrug resistance
MFP: mating pair formation
SEM: scanning electron microscopy
T4SS: type IV secretion system
WT: wild type
