# Supplementary material for: Mutation in ESBL Plasmid from Escherichia coli O104:H4 Leads Autoagglutination and Enhanced Plasmid Dissemination
Source: Front Microbiol. 2018 Feb 2;9:130. doi: 10.3389/fmicb.2018.00130 (PMC5801416; doi:10.3389/fmicb.2018.00130)
Supplement: Supplementary file 1 [file Presentation_1.PDF]

SUPPLEMENTAL MATERIALS

Legend to Fig. S1
Table S1 : Plasmids used in this study
Table S2 : Strains used in this study
Table S3 : Oligonucleotides used in this study
References for Supplemental Materials

FIG S1. Phenotypes of E. coli cells harboring conjugative plasmid.
(A, B and D) Sedimentation phenotype measured by OD 600nm of culture supernatant settled on bench for indicated time. Average of at least 3 independent experiments were shown. (C) Cell growth of *E. coli* strain harboring indicated plasmid (point mutants were color-coded as B) measured by OD 600 nm with plate reader. Average of at least 3 independent experiments were shown. (E) Transfer efficiency of indicated plasmid from Δ*fliA* donor cells. Average and standard deviation of 3 independent experiments were shown. (F) Representative FE-SEM image of *E. coli* cells harboring indicated plasmid. Pt-coated cells were imaged using secondary electrons. Scale bar = 1 µm. (G) Biofilm formation of *E. coli* cells harboring indicated plasmid, grown in LB (black bars) or M9 (gray bars) media. Average and standard deviations of at least 3 independent experiments were shown.

Table S1 : Plasmids used in this study

Plasmid	Description	Construction/Reference
pCP20	rep_{ts}, AmpR, CmR, *FRT*$^{+}$	Datsenko and Wanner (2000)
pDM4	rep_{R6K}, CmR, *sucB*	Milton et al. (1996)
pEYY39	pDM4 Hft and flanking region	PCR oYo40/oYo43, then Gibson Assembly with pDM4
pEYY40	pDM4 flanking regions of *oriT*	PCRs oYo107/oYo108, and oYo110/oYo111, then 3-piece Gibson Assembly with pDM4
pEYY45	pEYY39 X1	pEYY39 quickchange with oYo80/oYo81
pEYY70	pEYY39 X2	pEYY39 quickchange with oYo168/oYo169
pEYY71	pEYY39 X1X2	pEYY70 quickchange with oYo168/oYo169
pEYY72	pEYY39 X3	pEYY39 quickchange with oYo187/188
pEYY142	pEYY39 X1X2X3	pEYY71 quickchange with oYo187/188
pEYY143	pEYY39 X1X3	pEYY72 quickchange with oYo80/oYo81
pEYY144	pEYY39 X2X3	pEYY72 quickchange with oYo168/oYo169
pBAD33	P_{ara}, CmR	Guzman et al. (1995)
pEYY169	pBAD33 *traBC*	PCR oYo450/oYo451, then Gibson Assembly with pBAD33
pEYY192	pBAD33 *traA*	PCR oYo480/oYo481, then Gibson Assembly with pBAD33
pEYY207	pBAD33 *traA**	PCR oYo480/oYo481 from pESBL X3, then Gibson Assembly with pBAD33
pCB192-YY	AmpR, *lacZ*$^{+}$ (no EcoRI site in the 3' of *lacZ*)	Yamaichi et al. (2011)
pEYY167	pCB192 P1P2 (ii)~*traB::lacZ*	PCR oYo455/oYo454, then restriction cloning at HindIII-EcoRI site of pCB192-YY

pEYY168	pCB192 P1~*traB::lacZ*	PCR oYo455/oYo456, then restriction cloning at HindIII-EcoRI site of pCB192-YY
pEYY171	pCB192 P1P2 (i)~*traB::lacZ*	PCR oYo455/oYo461, then restriction cloning at HindIII-EcoRI site of pCB192-YY
pEYY176	pCB192 Hft~*traB::lacZ*	PCR oYo455/oYo470, then restriction cloning at HindIII-EcoRI site of pCB192-YY
pEYY177	pCB192 Hft X1~*traB::lacZ*	PCR oYo455/oYo470 from pESBL X1, then restriction cloning at HindIII-EcoRI site of pCB192-YY
pEYY178	pCB192 Hft X2~*traB::lacZ*	PCR oYo455/oYo470 from pESBL X2, then restriction cloning at HindIII-EcoRI site of pCB192-YY
pEYY179	pCB192 Hft X3~*traB::lacZ*	PCR oYo455/oYo470 from pESBL X3, then restriction cloning at HindIII-EcoRI site of pCB192-YY
pEYY180	pCB192 Hft X1X2~*traB::lacZ*	PCR oYo455/oYo470 from pESBL X1X2, then restriction cloning at HindIII-EcoRI site of pCB192-YY
pEYY181	pCB192 Hft X1X3~*traB::lacZ*	PCR oYo455/oYo470 from pESBL X1X3, then restriction cloning at HindIII-EcoRI site of pCB192-YY
pEYY182	pCB192 Hft X2X3~*traB::lacZ*	PCR oYo455/oYo470 from pESBL X2X3, then restriction cloning at HindIII-EcoRI site of pCB192-YY
pEYY183	pCB192 Hft X1X2X3~*traB::lacZ*	PCR oYo455/oYo470 from pESBL X1X2X3, restriction cloning at HindIII-EcoRI site of pCB192-YY
pEYY187	pCB192 Hft::Tn1Δkan~*traB::lacZ*	PCR oYo473/oYo474 from pESBL Tn, then Gibson Assembly with pCB192-YY
pEYY191	pCB192 ΔHft~*traB::lacZ*	PCR oYo455/oYo479, then restriction cloning at HindIII-EcoRI site of pCB192-YY
pEYY194	pCB192 Hft~*traA::lacZ*	PCR oYo473/oYo483, then Gibson Assembly with pCB192-YY
pEYY201	pCB192 Hft::Tn1Δkan~*traA::lacZ*	PCR oYo473/oYo483 from pESBL Tn, then Gibson Assembly with pCB192-YY
pEYY210	pCB192 Hft X1~*traA::lacZ*	PCR oYo473/oYo483 from pESBL X1, then Gibson Assembly with pCB192-YY
pEYY211	pCB192 Hft X2~*traA::lacZ*	PCR oYo473/oYo483 from pESBL X2, then Gibson Assembly with pCB192-YY
pEYY212	pCB192 Hft X1X2~*traA::lacZ*	PCR oYo473/oYo483 from pESBL X1X2, then Gibson Assembly with pCB192-YY
pEYY217	pCB192 ΔHft~*traA::lacZ*	Two oligo DNAs oYo547/oYo548 were annealed and cloned into HindIII-EcoRI site of pCB192-YY
pEYY269	pDM4 flanking regions of *pilS*	PCRs oYo645/oYo646, and oYo647/oYo648, then 3-piece Gibson Assembly with pDM4

Table S2 : Strains used in this study

Strain*	Description	Construction/Reference
BW25113†	F^{-}, DE*(araD-araB)567*, *lacZ4787(del)::rrnB-3*, LAM^{-}, *rph-1*, DE*(rhaD-rhaB)568*, *hsdR514*	Datsenko and Wanner (2000)
JW1907	BW25113 *ΔfliA::kan*	KEIO collection (Baba et al., 2006)
MC1061	K-12, SmR	laboratory stock
MKW278	MG1655 *ΔlacZ::cat*	Yamaichi et al. (2015)
	TN102 / R64 (Tet^{R}, Sm^{R})	Nobuhisa Furuya & Teruya Komano
	JA221 / R64 *drd11* (Tet^{R}, Sm^{R})	Nobuhisa Furuya & Teruya Komano
	MG1655 *rpsL* / F1-10 (Tet^{R})	Christian Lesterlin

YBB1195	MC1061 / pESBL (WT)	Yamaichi et al. (2015)
YBB1222	MC1061 / pESBL::Tn1	Yamaichi et al. (2015)
YBB1272	MC1061 / pESBL ΔHft	Yamaichi et al. (2015)
bEYY1097	MC1061 / pESBL::Tn1Δkan (hereafter referred to as Tn)	This study
bEYY1118	MC1061 / pESBL X1	This study
bEYY1193	MC1061 / pESBL X2	This study
bEYY1211	MC1061 / pESBL, pBAD33	This study
bEYY1216	MC1061 / pESBL ΔHft, pBAD33	This study
bEYY1195	MC1061 / pESBL X3	This study
bEYY1283	MC1061 / pESBL Tn Δ*oriT*	This study
bEYY1323	MC1061 *ΔfliA::kan*	This study
bEYY1324	MC1061 *ΔfliA::kan* / pESBL (WT)	This study
bEYY1325	MC1061 *ΔfliA::kan* / pESBL::TnBΔkan	This study
bEYY1391	MC1061 / pESBL X1X3	This study
bEYY1453	MC1061 / pESBL Hft::*kan rpsL*$^{+}$	This study
bEYY1481	MC1061 / pESBL, pEYY169	This study
bEYY1499	MC1061 Δ*fliA* / F1-10	This study
bEYY1501	MC1061 / pESBL X1X2X3	This study
bEYY1502	MC1061 / pESBL X2X3	This study
bEYY1527	MKW278 / R64	This study
bEYY1529	MKW278 / R64 *drd11*	This study
bEYY1549	MC1061 / pESBL Tn, pEYY169	This study
bEYY1550	MC1061 / pESBL ΔHft, pEYY169	This study
bEYY1557	MC1061 / pESBL Tn, pBAD33	This study
bEYY1574	MC1061 / pESBL, pEYY192	This study
bEYY1575	MC1061 / pESBL Tn, pEYY192	This study
bEYY1576	MC1061 / pESBL ΔHft, pEYY192	This study
bEYY1638	MC1061 / pESBL, pEYY207	This study
bEYY1639	MC1061 / pESBL Tn, pEYY207	This study
bEYY1640	MC1061 / pESBL ΔHft, pEYY207	This study
bEYY1666	MC1061 / F1-10	This study
bEYY1640	MC1061 / pESBL ΔHft, pEYY207	This study
bEYY1760	MC1061 / pESBL Δ*pilS*	This study
bEYY1761	MC1061 / pESBL Tn Δ*pilS*	This study
bEYY1778	MC1061 *ΔfliA::kan* / pESBL Δ*pilS*	This study
bEYY1779	MC1061 *ΔfliA::kan* / pESBL Tn Δ*pilS*	This study
bEYY1785	MC1061 / pESBL X3, pBAD33	This study
bEYY1786	MC1061 / pESBL X3, pEYY192	This study
bEYY1787	MC1061 / pESBL X3, pEYY207	This study
bEYY1788	MC1061 / pESBL X3, pEYY169	This study
PA14	*Pseudomonas aeruginosa* reference strain	Isabelle Vallet-Gely

*All strains except PA14 are *Escherichia. coli.*
†Host strain of the all β-galactosidase assay. BW25113 harboring different pCB192-derived plasmids were omitted from this list

Table S3 : Oligonucleotides used in this study

Name	Sequence (5'-3')*
oYo40	GCGGAGTGTATATCAAGCTTATCGCTCACAGATAAAGAACGGC
oYo43	TTGTGAGCGGATAACAATTTGTGGTCTTACAACCGATCAACC

oYo80	CAACTAAAAACGCTATCACCTAGTTCCTACGCTCATTGGTG
oYo81	CACCAATGAGCGTAGGAACTAGGTGATAGCGTTTTTAGTTG
oYo168	GCTAATGCCTGCCCCGCCTGATCTACCATAGATGACGACAAG
oYo169	CTTGTCGTCATCTATGGTAGATCAGGCGGGGCAGGCATTAGC
oYo170	GTAAGGTCGCCATATATCGG
oYo171	TTATACACTGCACAGGGTTG
oYo187	CAGATTTAAGTTCTTCAGGCTTCACTTCAGGTAGGTTCGTACCGTTTC
oYo188	GAAACGGTACGAACCTACCTGAAGTGAAGCCTGAAGAACTTAAATCTG
oYo417	TAAGTTTTGACGCTTTTTTGTATGTTTTTTAAGCGGTTAG TTGACTCGGTGGCCTGGTGATGATGGCGGGATCG
oYo421	ACAGCAGAGCGAAGTGCATCATATCCTTCCAGATTTAAGTTCTTCAG GCTTCAGAAGAACTCGTCAAGAAGGCG
oYo450	TGGGCTAGCGAATTCGAGCTGTGAATATAGAACACCTGAATAACCGGAACTG
oYo451	CCAAGCTTGCATGCCTGCAGTCAGTGTAATGTGATAGTCGTCTGCTCCT
oYo454	ATGAGCAAGCTTACAGGTGTCCCCATTAAAGGG
oYo455	ATGAGCGAATTCATATTCACGAAGTTCAACGCTCC
oYo456	ATGAGCAAGCTTAATAAAGCAAATCACTTCAGACGG
oYo461	ATGAGCAAGCTTCAGGCGCTCCCAAATTG
oYo470	ATGAGCAAGCTTTACGTTATACTAAGTACCC
oYo473	CCCGGGGATGGGGAGTAAGCTTTACGTTATACTAAGTACCC
oYo474	GACGTTGTAAAACGACGGCCAGTGAATTCATATTCACGAAGTTCAACGCTCC
oYo479	ATGAGCAAGCTTGTTGTCATCGGAGACCTTTGC
oYo483	GACGTTGTAAAACGACGGCCAGTGAATTCGGTGACATAGCAAAGGTCTCC
oYo547	AGCTTGTTGTCATCGGAGACCTTTGCTATGTCACCG
oYo548	AATTCGGTGACATAGCAAAGGTCTCCGATGACAACA
oYo645	GAGCGGATAACAATTTGTGGGCAGTGGTCACAGGTGTAATATCATG
oYo646	CATTAAAACTCCAAATACCTCGTTTTATTTAAAGATTACCCATACTGTTG
oYo647	ACGAGGTATTTGGAGTTTTAATGTCCGGGGGTAAAG
oYo648	GGAGTGTATATCAAGCTTATCGGTCACGATAAATTCTCCATACTTTATTAGCATATTGC

*5’ extensions including restriction sites and homology region for Gibson Assembly and λ Red recombination were indicated in gray. Nucleotide substitution to be introduced was indicated in red.

References for Supplemental Materials

Baba, T., Ara, T., Hasegawa, M., Takai, Y., Okumura, Y., Baba, M., et al. (2006). Construction of *Escherichia coli* K-12 in-frame, single-gene knockout mutants: the Keio collection. *Mol. Syst. Biol.* 2, 2006.0008.

Datsenko, K.A., and Wanner, B.L. (2000). One-step inactivation of chromosomal genes in *Escherichia coli* K-12 using PCR products. *Proc. Natl. Acad. Sci. U. S. A.* 97, 6640-6645.

Guzman, L.M., Belin, D., Carson, M.J., Beckwith, J. (1995). Tight regulation, modulation, and high-level expression by vectors containing the arabinose PBAD promoter. *J. Bacteriol.* 177, 4121-4130.

Milton, D.L., O'Toole, R., Horstedt, P., Wolf-Watz, H. (1996). Flagellin A is essential for the virulence of *Vibrio anguillarum*. *J. Bacteriol*. 178, 1310-1319.

Yamaichi, Y., Gerding, M.A., Davis, B.M., Waldor, M.K. (2011). Regulatory Cross-Talk Links *Vibrio cholerae* Chromosome II Replication and Segregation. *PLoS. Genet.* 7, e1002189.

Yamaichi, Y., Chao, M.C., Sasabe, J., Clark, L., Davis, B.M., Yamamoto, N., et al. (2015). High-resolution genetic analysis of the requirements for horizontal transmission of the ESBL plasmid from *Escherichia coli* O104:H4. *Nucleic. Acids. Res.* 43, 348-360.

Figure S1

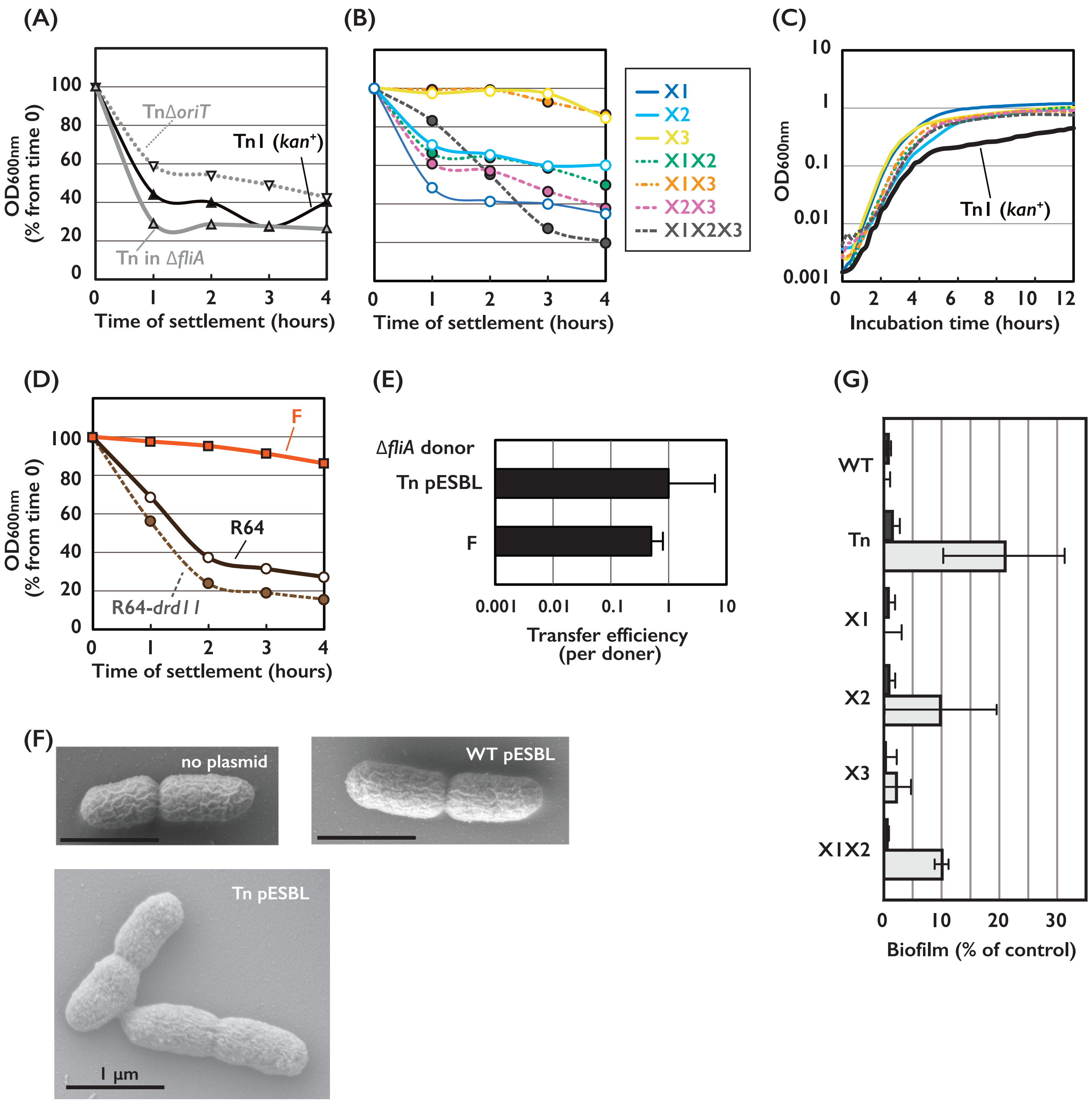

FIGURE S1. Phenotypes of E. coli cells harboring conjugative plasmid.

(A, B and D) Sedimentation phenotype measured by OD600nm of culture supernatant settled on bench for indicated time. Average of at least 3 independent experiments were shown. (C) Cell growth of E. coli strain harboring indicated plasmid (point mutants were color-coded as B) measured by OD600nm with plate reader. Average of at least 3 independent experiments were shown. (E) Transfer efficiency of indicated plasmid from ΔfliA donor cells. Average and standard deviation of 3 independent experiments were shown. (F) Representative FE-SEM image of E. coli cells harboring indicated plasmid. Pt-coated cells were imaged using secondary electrons. Scale bar = 1 μm. (G) Biofilm formation of E. coli cells harboring indicated plasmid, grown in LB (black bars) or M9 (gray bars) media. Average and standard deviations of at least 3 independent experiments were shown.
